# Phase 1 study of the MDM2 inhibitor AMG 232 in patients with advanced P53 wild-type solid tumors or multiple myeloma

**DOI:** 10.1007/s10637-019-00840-1

**Published:** 2019-07-29

**Authors:** W. Larry Gluck, Mrinal M. Gounder, Richard Frank, Ferry Eskens, Jean Yves Blay, Philippe A. Cassier, Jean-Charles Soria, Sant Chawla, Vincent de Weger, Andrew J. Wagner, David Siegel, Filip De Vos, Erik Rasmussen, Haby A. Henary

**Affiliations:** 1 Prisma Health - Upstate, Institute for Translational Oncology Research, 900 W. Faris Rd., 3rd Floor, Greenville, SC 29605 USA; 2 Department of Medicine, Memorial Sloan-Kettering Cancer Center, New York, NY USA; 3 Whittingham Cancer Center, Norwalk, CT USA; 4 Medical Oncology, Erasmus MC Cancer Institute, Rotterdam, The Netherlands; 5 Department of Medicine, Centre Léon Bérard, Lyon, France; 6 Department of Medicine, The Institute Gustave-Roussy, Paris, France; 7 Université Paris Sud, Orsay, France; 8 Sarcoma Oncology Center, Cancer Center of Southern California, Santa Monica, CA USA; 9 Department of Internal Medicine, Netherlands Cancer Institute, Amsterdam, The Netherlands; 10 Center for Sarcoma and Bone Oncology and Department of Medical Oncology, Dana-Farber Cancer Institute, Harvard Medical School, Boston, MA USA; 11 Multiple Myeloma Division, John Theurer Cancer Center at the Hackensack University Medical Center, Hackensack, NJ USA; 12 Medical Oncology, University Medical Center Utrecht, Utrecht University, Utrecht, The Netherlands; 13 Oncology Early Development, Amgen Inc., Thousand Oaks, CA USA

**Keywords:** AMG 232, MDM2, MDM2 inhibitor, Phase 1 trial, Solid tumors, Multiple myeloma

## Abstract

*Background* This open-label, first-in-human, phase 1 study evaluated AMG 232, an oral selective MDM2 inhibitor in patients with *TP53* wild-type (P53WT), advanced solid tumors or multiple myeloma (MM). *Methods* In the dose escalation (*n* = 39), patients with P53WT refractory solid tumors enrolled to receive once-daily AMG 232 (15, 30, 60, 120, 240, 480, and 960 mg) for seven days every 3 weeks (Q3W). In the dose expansion (*n* = 68), patients with *MDM2*-amplified (well-differentiated and de-differentiated liposarcomas [WDLPS and DDLPS], glioblastoma multiforme [GBM], or other solid tumors [OST]), MDM2-overexpressing ER+ breast cancer (BC), or MM received AMG 232 at the maximum tolerated dose (MTD). Safety, pharmacokinetics, pharmacodynamics, and efficacy were assessed. *Results* AMG 232 had acceptable safety up to up to 240 mg. Three patients had dose-limiting toxicities of thrombocytopenia (*n* = 2) and neutropenia (*n* = 1). Due to these and other delayed cytopenias, AMG 232 240 mg Q3W was determined as the highest tolerable dose assessed in the dose expansion. Adverse events were typically mild/moderate and included diarrhea, nausea, vomiting, fatigue, decreased appetite, and anemia. AMG 232 plasma concentrations increased dose proportionally. Increases in serum macrophage inhibitor cytokine-1 from baseline were generally dose dependent, indicating p53 pathway activation. Per local review, there were no responses. Stable disease (durability in months) was observed in patients with WDLPS (3.9), OST (3.3), DDLPS (2.0), GBM (1.8), and BC (1.4–2.0). *Conclusions* In patients with P53WT advanced solid tumors or MM, AMG 232 showed acceptable safety and dose-proportional pharmacokinetics, and stable disease was observed.

## Introduction

The tumor suppressor p53 is a key regulator of cell cycle progression and apoptosis [1, 2]. Mouse double minute 2 homolog (MDM2) inhibits p53 activity by acting as an E3 ubiquitin ligase to promote its degradation, by binding and blocking the p53 transcriptional activation domain, and by exporting p53 from the nucleus to the cytoplasm [3, 4]. Among *TP53* wild-type (P53WT) solid tumors, *MDM2* amplification has been demonstrated in well-differentiated and de-differentiated liposarcomas (WDLPS and DDLPS, respectively) and in glioblastoma multiforme (GBM) [5, 6]. Overexpression of MDM2 protein has been shown in WDLPS and DDLPS, estrogen receptor positive (ER+) breast cancer, and multiple myeloma [7–11]. *MDM2* amplification and *MDM2* overexpression, which result in p53 inactivation and decreased apoptosis, have been associated with poor outcomes [12–14].

Although many tumors harbor non-targetable mutations in *TP53*, MDM2 has become an attractive therapeutic target in the treatment of *TP53* wild-type (P53WT) cancers. Several MDM2 inhibitors are in clinical investigation as monotherapy or combined with other therapies for the treatment of P53WT hematologic malignancies and solid tumors [15–18]. In clinical studies of MDM2 inhibitors, increases in circulating macrophage inhibitor cytokine-1 (MIC-1) has been used as a pharmacodynamic marker of p53 activation [19–21].

AMG 232 is an investigational oral, selective MDM2 inhibitor that restores p53 tumor suppression by blocking the MDM2-p53 interaction with picomolar affinity [22]. In tumor xenograft models, treatment with AMG 232 resulted in tumor growth inhibition and caused regression of *MDM2*-amplified tumors through the induction of growth arrest and apoptosis [23]. The primary objectives of this open-label, first-in-human, phase 1 study were to assess the safety and tolerability, pharmacokinetics, maximum tolerated dose (MTD), pharmacodynamics, and efficacy of AMG 232 in patients with P53WT solid tumors or multiple myeloma.

## Methods

### Patients

Patients aged ≥18 years with pathologically-documented, P53WT (per next-generation sequencing) treatment-refractory solid tumors measurable per Response Evaluation Criteria in Solid Tumors (RECIST) version 1.1 or Macdonald criteria for glioblastoma multiforme (GBM), or progressive multiple myeloma measurable per International Myeloma Working Group (IMWG) response criteria were eligible. Additional eligibility criteria were Eastern Cooperative Oncology Group (ECOG) performance status ≤2; life expectancy >3 months; adequate hematologic (ANC ≥1.5 × 10^9^/L for solid tumors or ≥ 1.0 × 10^9^/L for multiple myeloma; platelet count ≥100 × 10^9^/L for solid tumors or ≥ 75 × 10^9^/L for multiple myeloma; hemoglobin >9 g/dL), renal (estimated glomerular filtration rate ≥ 45 mL/min/1.73 m^2^), hepatic (AST and AST <2.5 × ULN; ALP <2.0 × ULN; total bilirubin <1.5 × ULN), and coagulation (prothrombin time or partial thromboplastin time < 1.5 × ULN) functions. In the dose expansion, five tumor types were defined: well-differentiated or dedifferentiated liposarcoma; relapsed GBM with *MDM2* amplification; estrogen receptor positive (ER+) breast cancer refractory to hormonal treatments; relapsed multiple myeloma progressive after ≥1 prior treatment; other advanced solid tumors with *MDM2* amplification. Key exclusion criteria included active or untreated brain metastases; unresolved toxicity from prior anticancer therapy, excluding alopecia; antitumor therapy or major surgery within 28 days of starting study treatment; investigational device or drug within 30 days or 5 half-lives of starting study treatment; liposarcomas with >3 prior approved therapies; multiple myeloma with del (17p) or IgM subtype, non-secretory or hyposecretory disease, lack of ≥25% reduction in M-protein for ≥6 weeks with prior therapy, corticosteroid therapy within 3 weeks of study, POEMS syndrome, or plasma cell leukemia or lymphoplasmacytic lymphoma. Institutional review board approval was obtained for all study procedures. All patients provided informed consent before enrollment.

### Study design and treatment

This open-label phase 1 study was conducted at 16 centers (ClinicalTrials.gov, NCT01723020). *TP53* mutation status was confirmed by central laboratory assessment. The study was planned with two parts: a 3-part dose escalation (Part 1) and a dose expansion (Part 2). In the dose escalation, multiple-patient cohorts were planned to enroll sequentially (Part 1A) or in parallel (Parts 1B and 1C; 3 + 3 design) and to receive AMG 232 once daily (QD) for 3 days (Part 1B) or 7 days (Part 1C) every 3 weeks (Q3W) at prespecified doses of 15, 30, 60, 120, 240, 480, and 960 mg. Intermediate doses were allowed when deemed appropriate. The dose expansion was planned for patients with *MDM2-*amplified tumors (group 1; liposarcomas, GBM, and other solid tumors), potentially MDM2-overexpressing tumors (group 2; ER+ metastatic breast cancer), or multiple myeloma (group 3).

Each patient was monitored for 21 days for the occurrence of dose-limiting toxicities (DLTs), defined as febrile neutropenia, neutropenic infection, grade 4 neutropenia lasting >7 days, grade ≥ 3 thrombocytopenia lasting >7 days (solid tumors only), grade 3 thrombocytopenia with grade ≥ 2 bleeding (solid tumors only), grade 3 or 4 thrombocytopenia with grade > 1 bleeding (multiple myeloma only), grade 4 thrombocytopenia (solid tumors only), or grade 4 thrombocytopenia lasting >14 days (multiple myeloma only), or as grade ≥ 3 nausea, vomiting, or diarrhea after support; grade 3 fatigue lasting >7 days; any other grade ≥ 3 adverse event (AE); grade ≥ 3 kidney injury (multiple myeloma only), or treatment-related AEs not returning to grade ≤ 1 (solid tumors only) per Common Terminology Criteria for Adverse Events (CTCAE), version 4.0. The MTD was defined as the maximum dose, at which the probability of a DLT was ≤25% in Part 1A and ≤ 33% in Parts 1B and 1C. Treatment continued until disease progression, intolerable toxicity, or withdrawal of consent.

### Study assessments

#### Safety

AEs were recorded for all enrolled patients.

#### Pharmacokinetics

Plasma samples for the measurement of AMG 232 pharmacokinetics in Parts 1 and 2 were collected predose and at 1, 3, 5, and 7 h postdose on days 1 and 7 and 24 and 72 h postdose from day 7 of cycle 1; predose on days 1 and 7 of cycle 2; and at the end of study. Plasma AMG 232 levels were measured using a validated high performance liquid chromatography mass spectrometry method [24]. Pharmacokinetic and exposure parameters were estimated, including terminal half-life (t_max_), maximum observed plasma concentration (C_max_), area under the concentration-versus-time curve at 24 h (AUC_24h_), volume of distribution (V_z_/F), terminal elimination half-life (t_1/2,z_), and clearance (CL/F). Non-compartmental analysis was performed using WinNonlin Professional software, version 6.3. Parameters were summarized descriptively.

#### Circulating MIC-1

In Parts 1 and 2, serum samples for the assessment of circulating macrophage inhibitor cytokine-1 (MIC-1) were collected on the pharmacokinetic sample schedule. Serum MIC-1 concentrations were measured using a validated ELISA (human GDF-15 Quantikine®, R&D Systems Inc.).

#### Efficacy

Efficacy response was assessed using revised Response Evaluation Criteria in Solid Tumors (RECIST), version 1.1 [25] Macdonald criteria for GBM [26] or International Myeloma Working Group (IMWG) response criteria for multiple myeloma [27].

### Statistical analysis

Primary endpoints were the patient incidence of AEs, DLTs, and clinically significant changes in safety assessments; AMG 232 and pharmacokinetic parameters; and the MTD in Part 1. Secondary/exploratory endpoints included tumor response and change in serum MIC-1 level. Data were summarized descriptively. Qualified researchers may request data from Amgen clinical studies. Complete details are available at the following: http://www.amgen.com/datasharing.

## Results

### Patients

Overall, 107 patients enrolled (dose escalation, *n* = 39; dose expansion, *n* = 68). Patients in the dose escalation had a variety of refractory advanced solid tumors, and those in the dose expansion had WDLPS (*n* = 10), DDLPS (n = 10), GBM (n = 10), other solid tumors (*n* = 16), breast cancer (*n* = 12), or multiple myeloma (n = 10; Table 1). Most patients had received 3 or more lines of therapy (dose escalation, 69%; dose expansion, 72%).

**Table 1:** Demographics and baseline characteristics

Characteristics	Dose Escalation (n = 39)	Dose Expansion (n = 68)
Median (range) age, years	64 (41–84)	64 (36–82)
Sex, n (%)		
Men Women	26 (67) 13 (33)	27 (40) 41 (60)
Race, n (%)		
White Black Asian Other	34 (87) 3 (8) 2 (5) 0	60 (88) 2 (3) 5 (7) 1 (1)
Primary tumor type, n (%)		
Soft tissue sarcoma Liposarcoma Breast carcinoma Multiple myeloma Glioblastoma multiforme Colon carcinoma Non–small-cell lung carcinoma Head and neck carcinoma Thyroid carcinoma Pancreatic carcinoma Melanoma Salivary gland carcinoma Other	9 (23) 0 1 (3) 0 0 4 (10) 4 (10) 2 (5) 2 (5) 2 (5) 2 (5) 3 (8) 10 (26) ^a^	19 (28) 5 (7) 12 (18) 10 (15) 10 (15) 1 (1) 2 (3) 0 0 1 (1) 0 2 (3) 11 (16) ^b^
ECOG performance status, n (%)		
0 1 2	14 (36) 23 (59) 2 (5)	16 (24) 48 (71) 4 (6)
Prior lines of anticancer therapy, n (%)		
0 1 2 ≥3	2 (5) 2 (5) 8 (21) 27 (69)	6 (9) 4 (6) 9 (13) 49 (72)
Prior lines of radiotherapy, n (%)		
0 1 2 ≥3	17 (44) 9 (23) 6 (15) 7 (18)	29 (43) 25 (37) 9 (13) 5 (7)

ECOG, Eastern Cooperative Oncology Group

^a^Includes renal cell tumor (*n* = 2) and *n* = 1 each of mesothelioma, neuroendocrine cancer, rectal carcinoma, prostate cancer, neuroendocrine carcinoid, cholangiocarcinoma, esophageal cancer, and granular cell tumor

^b^Includes unknown (n = 2) and n = 1 each of bone tumor, cardia carcinoma, cholangiocarcinoma, endometrial cancer, ileal cancer, osteosarcoma, prostate cancer, squamous lung cancer, and renal cell cancer

AMG 232 was administered to all 39 patients in the dose escalation (15 mg, *n* = 3; 30 mg, n = 3; 60 mg, *n* = 4; 120 mg, *n* = 7; 240 mg, *n* = 8; 300 mg, n = 4; 360 mg, n = 4; 480 mg, *n* = 6) and to all 68 patients in the dose expansion (240 mg). The intermediate doses of 300 mg and 360 mg were assessed due to the occurrence of AEs. Reasons for discontinuing treatment across the entire study were disease progression (*n* = 74), AEs (*n* = 21), patient request (*n* = 11), and unknown (n = 1).

### Safety and tolerability

#### DLTs and MTD

Three patients in the dose escalation had DLTs. The first patient (120-mg cohort) with esophageal cancer had a DLT consisting of grade 3 thrombocytopenia on day 15 that worsened to grade 4 on day 17, lasted seven days, and required a platelet transfusion. The second patient (360-mg cohort) with rectal cancer had a DLT consisting of grade 4 thrombocytopenia on day 28. The third patient (480-mg cohort) with head and neck cancer had grade 3 neutropenia on day 22 that delayed treatment in the next cycle and was therefore considered a DLT. Two additional patients in the 300-mg cohort had cytopenias (grade 4 neutropenia; grade 4 thrombocytopenia) outside of the 21-day DLT evaluation window that were considered in the dose escalation decisions. Based on the protocol-specified definition using DLTs incidence, the MTD for AMG 232 for 7 days Q3W was not reached. However, when the DLTs and delayed cytopenias were considered, the highest safe and tolerable dose of AMG 232 was 240 mg, which was the dose evaluated in the dose expansion.

#### Safety and tolerability in the dose escalation

In the dose escalation, 37 (95%) patients had treatment-emergent AEs (Table 2), most of which were grade 1 or 2. The most common (occurring in ≥20% of patients) treatment-emergent AEs were diarrhea (67%), nausea (59%), vomiting, (51%), fatigue (41%), decreased appetite (39%), thrombocytopenia (36%), anemia (26%), neutropenia (26%), and abdominal pain (21%). Thirty-four (87%) patients in the dose escalation had AEs that were considered by the investigators to be treatment related. The most common (occurring in ≥20% of patients) treatment-related AEs were diarrhea (64%), nausea (51%), vomiting (46%), fatigue (41%), thrombocytopenia (36%), decreased appetite (26%), and neutropenia (21%). Most treatment-related AEs were grade 1 or 2.

**Table 2:** Patient incidence of adverse events in the AMG 232 dose escalation

	AMG 232 Dose Escalation Cohort								
	15 mg (n = 3)	30 mg (*n* = 3)	60 mg (*n* = 4)	120 mg (*n* = 7)	240 mg (*n* = 8)	300 mg (n = 4)	360 mg (n = 4)	480 mg (*n* = 6)	Total (*n* = 39)
Patients with any treatment-emergent AE, n (%)	1 (33)	3 (100)	4 (100)	7 (100)	8 (100)	4 (100)	4 (100)	6 (100)	37 (95)
Patients with any treatment-emergent serious AE, n (%)	1 (33)	0	0	3 (43)	4 (50)	2 (50)	0	4 (67)	14 (36)
Patients with any treatment-related AE, n (%)	1 (33)	1 (33)	3 (75)	7 (100)	8 (100)	4 (100)	4 (100)	6 (100)	34 (87)
Grade 3	0	0	0	1 (14)	3 (38)	3 (75)	3 (75)	3 (50)	13 (33)
Grade 4	0	0	0	1 (14)	0	2 (50)	2 (50)	3 (50)	8 (21)
Grade 5	0	0	0	0	0	0	0	0	0
Treatment-related AEs occurring in ≥10% of patients, n (%)									
Diarrhea	0	1 (33)	1 (25)	3 (43)	6 (75)	4 (100)	4 (100)	6 (100)	25 (64)
Nausea	0	1 (33)	3 (75)	3 (43)	5 (63)	1 (25)	4 (100)	3 (50)	20 (51)
Vomiting	0	1 (33)	0	2 (29)	5 (63)	3 (75)	3 (75)	4 (67)	18 (46)
Fatigue	1 (33)	1 (33)	1 (25)	3 (43)	4 (50)	2 (50)	0	4 (67)	16 (41)
Thrombocytopenia	0	0	0	1 (14)	1 (13)	3 (75)	4 (100)	5 (83)	14 (36)
Decrease appetite	0	0	1 (25)	2 (29)	2 (25)	1 (25)	3 (75)	1 (17)	10 (26)
Neutropenia	0	0	0	0	0	2 (50)	3 (75)	3 (50)	8 (21)
Anemia	0	0	0	0	1 (13)	0	2 (50)	2 (33)	5 (13)
Myalgia	0	0	0	1 (14)	0	0	2 (50)	1 (17)	4 (10)
Asthenia	0	0	0	0	1 (13)	1 (25)	2 (50)	0	4 (10)
Abdominal pain	0	0	0	0	2 (25)	1 (25)	1 (25)	0	4 (10)
Dysgeusia	0	0	0	0	2 (25)	1 (25)	1 (25)	0	4 (10)
Upper abdominal pain	0	0	0	1 (14)	1 (13)	1 (25)	1 (25)	0	4 (10)
Patients with any treatment-related serious AE, n (%)	0	0	0	0	2 (25)	2 (50)	0	2 (33)	6 (15)
Vomiting	0	0	0	0	1 (13)	2 (50)	0	0	3 (8)
Grade 2	0	0	0	0	0	1 (25)	0	0	1 (3)
Grade 3	0	0	0	0	1 (13)	1 (25)	0	0	2 (5)
Diarrhea	0	0	0	0	1 (13)	1 (25)	0	0	2 (5)
Grade 2	0	0	0	0	1 (13)	0	0	0	1 (3)
Grade 3	0	0	0	0	0	1 (25)	0	0	1 (3)
Thrombocytopenia (grade 4)	0	0	0	0	0	0	0	2 (33)	2 (5)
Hematemesis (grade 3)	0	0	0	0	0	0	0	1 (17)	1 (3)
Neutropenia (grade 4)	0	0	0	0	0	0	0	1 (17)	1 (3)
Dehydration (grade 2)	0	0	0	0	0	1 (25)	0	0	1 (3)
Febrile neutropenia (grade 3)	0	0	0	0	0	1 (25)	0	0	1 (3)
Abdominal pain (grade 2)	0	0	0	0	1 (13)	0	0	0	1 (3)
Nausea (grade 3)	0	0	0	0	1 (13)	0	0	0	1 (3)
Non-cardiac chest pain (grade 3)	0	0	0	0	1 (13)	0	0	0	1 (3)

AE, adverse event

Serious AEs occurred in 14 (36%) patients during the dose escalation, including six (15%) whose serious AEs were considered treatment-related and predominantly included gastrointestinal toxicity (Table 2). Overall, eight patients in the dose escalation had AEs resulting in treatment discontinuation: thrombocytopenia (*n* = 5), neutropenia (*n* = 2), and febrile neutropenia (*n* = 1). Four patients in the dose escalation had fatal AEs of disease progression while on study.

#### Safety and tolerability in the dose expansion

In the 240-mg dose expansion, 67 (99%) patients had treatment-emergent AEs (Table 3), most of which were grade 1 or 2. The most common (occurring in ≥20% of patients) treatment-emergent AEs were diarrhea (72%), nausea (72%), vomiting, (59%), fatigue (53%), decreased appetite (41%), anemia (25%), and thrombocytopenia (24%). Sixty-five (96%) patients in the dose expansion had AEs that were considered by the investigators to be related to treatment with AMG 232. The most common (occurring in ≥20% of patients) treatment-related AEs were diarrhea (68%), nausea (68%), vomiting (47%), fatigue (47%), decreased appetite (41%), and thrombocytopenia (21%). Most treatment-related AEs were grade 1 or 2.

**Table 3:** Patient incidence of adverse events in the AMG 232 dose expansion

	WDLPS (n = 10)	DDLPS (n = 10)	GBM (n = 10)	Other Solid (n = 16)	ER + PR+ Breast (n = 8)	ER + PR–Breast (n = 4)	Multiple Myeloma (n = 10)	Total (n = 68)
Patients with any treatment-emergent AE, n (%)	10 (100)	9 (90)	10 (100)	16 (100)	8 (100)	4 (100)	10 (100)	67 (99)
Patients with any treatment-emergent serious AE, n (%)	5 (50)	4 (40)	7 (70)	5 (31)	3 (38)	1 (25)	4 (40)	29 (43)
Patients with any treatment-related AE, n (%)	10 (100)	9 (90)	10 (100)	14 (88)	8 (100)	4 (100)	10 (100)	65 (96)
Grade 3	6 (60)	0	4 (40)	4 (25)	4 (50)	1 (25)	6 (60)	25 (37)
Grade 4	3 (30)	0	0	2 (13)	0	0	3 (30)	8 (12)
Grade 5	0	0	0	0	0	0	0	0
Treatment-related AEs occurring in ≥10% of patients, n (%)								
Diarrhea	9 (90)	6 (60)	5 (50)	8 (50)	7 (88)	4 (100)	7 (70)	46 (68)
Nausea	10 (100)	8 (80)	4 (40)	11 (69)	6 (75)	3 (75)	4 (40)	46 (68)
Vomiting	7 (70)	2 (20)	3 (30)	8 (50)	6 (75)	2 (50)	4 (40)	32 (47)
Fatigue	8 (80)	7 (70)	6 (60)	6 (38)	3 (38)	0	2 (20)	32 (47)
Decrease appetite	7 (70)	4 (40)	1 (10)	4 (25)	5 (63)	3 (75)	4 (40)	28 (41)
Thrombocytopenia	6 (60)	1 (10)	2 (20)	2 (13)	0	0	3 (30)	14 (21)
Neutropenia	1 (60)	0	2 (20)	1 (6)	0	0	3 (30)	12 (18)
Anemia	5 (50)	0	0	3 (19)	0	1 (25)	1 (10)	10 (15)
Asthenia	0	1 (10)	0	2 (13)	1 (13)	1 (25)	4 (40)	9 (13)
Dysgeusia	1 (10)	1 (10)	1 (10)	2 (13)	2 (25)	0	2 (20)	9 (13)
Patients with any serious, treatment-related AE, n (%)	2 (20)	0	1 (10)	0	2 (25)	0	2 (20)	7 (10)
Vomiting (grade 3)	0	0	0	0	1 (13)	0	2 (20)	3 (4)
Diarrhea (grade 3)	0	0	0	0	1 (13)	0	0	1 (2)
Nausea (grade 3)	0	0	0	0	1 (13)	0	0	1 (2)
Hyperamylasemia (grade 3)	0	0	1 (10)	0	0	0	0	1 (2)
Hyperlipasemia (grade 3)	0	0	1 (10)	0	0	0	0	1 (2)
Dehydration (grade 3)	1 (10)	0	0	0	0	0	0	1 (2)
Pulmonary embolism (grade 3)	1 (10)	0	0	0	0	0	0	1 (2)

AE, adverse event; DD, Dedifferentiated liposarcoma; WD, well differentiated liposarcoma

During the dose expansion, 29 (43%) patients had serious AEs, including seven (10%) whose serious AEs were considered treatment-related (Table 3). Overall, 13 (19%) patients in the dose expansion had AEs resulting in treatment discontinuation: vomiting (*n* = 4), fatigue (*n* = 2), and nausea, dyspnea, pulmonary embolism, asthenia, malaise, ECOG performance status 4, thrombocytopenia, neutropenia, intestinal adhesion lysis, and oculogyric crisis (*n* = 1 each). Three patients in the dose expansion had fatal AEs while on study, including two with disease progression and one with physical deterioration.

### Pharmacokinetics of AMG 232

**Fig. 1:**
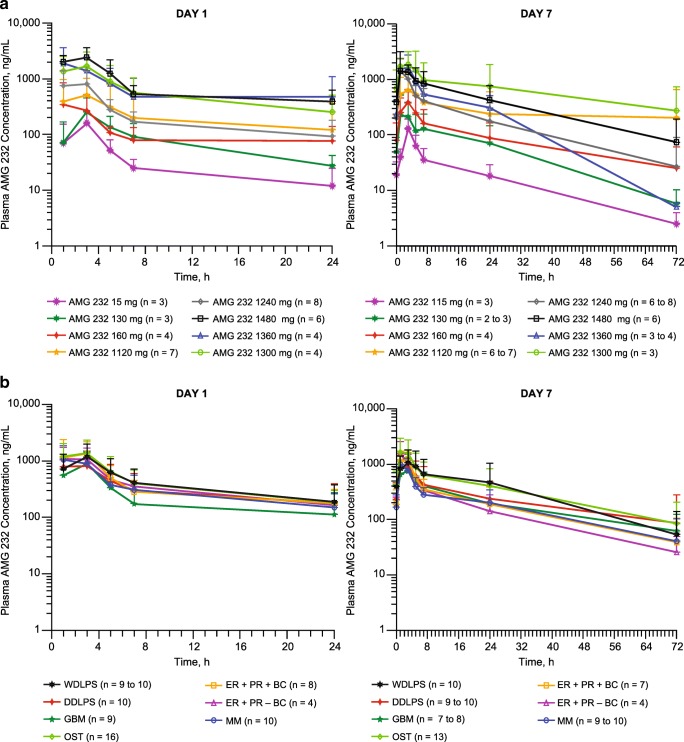
Mean (± SD) pharmacokinetic profile of AMG 232 following oral administration every 3 weeks in the dose escalation (**a**) and in the dose expansion (**b**). DDLPS, dedifferentiated liposarcoma; GBM, glioblastoma multiforme; OST, other solid tumor; WDLPS, well differentiated liposarcoma

Plasma samples for the evaluation of AMG 232 pharmacokinetics were available for 106 patients. AMG 232 pharmacokinetic profiles for the dose escalation and dose expansion are shown in Fig. 1. Plasma concentrations of AMG 232 increased dose proportionally. The mean AUC accumulation ratio between days 1 and day 7 across all dosing groups in the dose escalation and in the dose expansion was less than 2-fold with the once-daily dosing regimen (Table 4**,** Table 5). The mean estimated apparent volume of distribution was 615 L across all dosing cohorts. With oral administration, the estimated mean apparent clearance of AMG 232 was 30.2 L/h across groups and varied among individuals.

**Table 4:** AMG 232 pharmacokinetic parameters in the dose escalation

	Dose Escalation (Part 1)								Parts 1 and 2	
Characteristic^a^	15 mg (n = 3)	30 mg (n = 3)	60 mg (n = 2–4)	120 mg (n = 4–7)	240 mg (n = 7–8)	300 mg (n = 3–4)	360 mg (n = 3–4)	480 mg (n = 5–6)	240 mg (*n* = 54–75)	All Doses (*n* = 76–97)
Day 1										
C_max_, ng/mL	169 (100)	255 (198)	527 (428)	599 (585)	1030 (603)	1740 (1240)	2380 (1560)	2630 (977)	1350 (785)	–
t_max_, h	3.1 (1.1–3.3)	3.0 (3.0–3.1)	3.0 (1.1–24)	3.0 (1.0–3.1)	2.0 (1.0–3.1)	3.0 (1.0–3.1)	2.0 (0.93–3.2)	3.0 (1.0–3.3)	3.0 (0.98–24)	–
AUC_24h_, ng•h/mL	884 (482)	1960 (1230)	2950 (785)	5160 (8170)	5690 (3180)	14,700 (12,300)	15,800 (13,900)	18,800 (5970)	8480 (5280)	–
Day 7										
C_max_, ng/mL	130 (64)	259 (211)	457 (277)	868 (816)	1560 (1460)	2090 (1190)	2050 (1080)	1750 (501)	1440 (1020)	–
t_max_, h	3.1 (2.4–3.3)	3.0 (1.0–3.3)	4.0 (1.1–7.0)	3.0 (1.0–5.0)	3.0 (1.1–7.0)	3.1 (0.13–5.3)	2.0 (1.0–3.3)	3.1 (1.0–7.1)	2.9 (0.92–7.0)	–
AUC_24h_, ng•h/mL	949 (489)	2830 (2460)	4000 (2710)	8940 (12,000)	10,600 (5550)	25,000 (26,300)	15,500 (6560)	18,400 (10,100)	12,100 (9160)	–
CL/F, L/h	20.9 (15.1)	27.8 (33.7)	21.6 (15.2)	31.1 (19.6)	30.7 (19.5)	26.9 (25.0)	27.6 (17.4)	35.2 (25.6)	31.0 (20.6)	30.2 (20.4)
V_z_/F, L	497 (336)	1110 (1660)	360 (19.9)	664 (324)	523 (404)	762 (584)	423 (386)	585 (308)	613 (452)	615 (500)
t_1/2,z_, h	16.7 (1.4)	19.2 (10.7)	14.3 (1.8)	12.4 (0.6)	12.4 (8.4)	13.3 (2.3)	9.5 (1.6)	13.7 (7.5)	14.0 (6.2)	14.0 (6.0)
AUC_24h_ AR	1.10 (0.37)	1.38 (1.13)	1.76 (1.04)	2.63 (2.47)	2.10 (1.14)	2.42 (1.69)	1.29 (0.59)	0.95 (0.34)	1.49 (0.80)	1.58 (1.07)

AR, accumulation ratio (AUC_24h cycle 1, day 1_ / AUC_24h cycle 1, day 7_); AUC_24h_, area under the concentration-versus-time curve at 24 h; CL/F, clearance; C_max_, maximum observed serum concentration; t_1/2,z_, terminal elimination half-life; t_max_, time to reach C_max_; V_z_/F, volume of distribution

^a^All data are mean (SD) except for t_max_, which is median (range)

**Table 5:** AMG 232 pharmacokinetic parameters in the 240-mg dose expansion

Characteristic^a^	WDLPS (n = 9–10)	DDLPS (n = 6–10)	GBM (n = 6–9)	Other Solid (*n* = 9–16)	ER + PR+ Breast (*n* = 6–8)	ER + PR– Breast (n = 4)	Multiple Myeloma (n = 7–10)	Total (*n* = 47–67)
Day 1								
C_max_, ng/mL	1350 (815)	986 (582)	978 (630)	1670 (858)	1920 (1010)	1360 (487)	1320 (687)	1390 (798)
t_max_, h	3.1 (1.1–3.1)	2.0 (0.98–3.1)	3.0 (1.0–3.3)	3.0 (0.98–5.2)	2.8 (1.0–24)	2.1 (1.0–3.1)	2.0 (1.0–3.1)	3.0 (0.98–24)
AUC_24h_, ng•h/mL	9260 (5500)	8010 (6760)	5360 (3650)	10,800 (6270)	9660 (3890)	9430 (4480)	8220 (4790)	8830 (5400)
Day 7								
C_max_, ng/mL	1230 (803)	1050 (660)	988 (591)	2200 (1400)	1350 (587)	1620 (923)	1190 (605)	1420 (961)
t_max_, h	3.0 (1.0–5.1)	3.0 (1.0–3.1)	2.8 (1.0–5.0)	2.9 (0.92–5.0)	2.8 (1.1–5.1)	1.0 (1.0–3.0)	1.1 (1.0–3.4)	2.8 (0.92–5.1)
AUC_24h_, ng•h/mL	15,700 (13,800)	10,800 (9640)	9210 (6400)	17,200 (11,400)	10,400 (4380)	9960 (7830)	8450 (3560)	12,300 (9550)
CL/F, L/h	30.6 (24.1)	39.5 (29.0)	41.2 (27.5)	18.5 (9.1)	27.6 (12.6)	36.4 (22.8)	33.6 (15.4)	31.1 (20.9)
V_z_/F, L	389 (237)	537 (435)	1010 (671)	353 (360)	689 (529)	814 (455)	859 (277)	627 (461)
t_1/2,z_, h	11.2 (4.3)	10.9 (4.3)	18.5 (5.3)	14.1 (7.6)	15.0 (6.3)	17.4 (6.9)	15.4 (3.5)	14.3 (5.8)
AUC_24h_ AR	1.62 (0.95)	1.43 (0.72)	1.50 (0.31)	1.52 (0.57)	1.01 (0.25)	0.99 (0.43)	1.39 (1.05)	1.40 (0.72)

AR, accumulation ratio (AUC_24h cycle 1, day 1_ / AUC_24h cycle 1, day 7_); AUC_24h_, area under the concentration-versus-time curve at 24 h; CL/F, clearance; C_max_, maximum observed serum concentration; t_1/2,z_, terminal elimination half-life; t_max_, time to reach C_max_; V_z_/F, volume of distribution

^a^All data are mean (SD) except for t_max_, which is median (range)

### AMG 232 pharmacodynamic effects

**Fig. 2:**
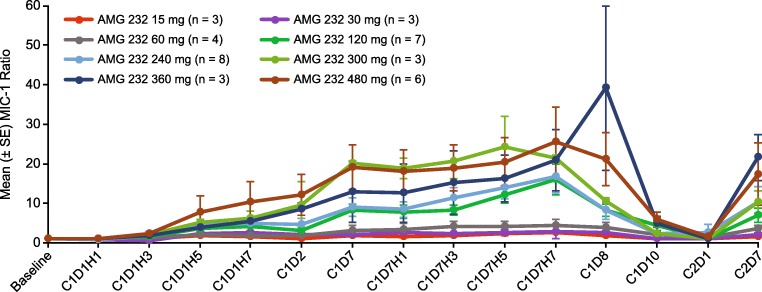
Mean (± SE) ratio of post-treatment versus pre-treatment serum MIC-1 in the dose escalation

Thirty-nine patients in the dose escalation had available pre-treatment and post-treatment blood samples for the assessment of serum MIC-1; serum MIC- was not assessed in the dose expansion. From baseline to day 15, increases in serum MIC-1 (post-treatment to pre-treatment ratios) were generally dose-dependent (Fig. 2). Mean serum MIC-1 ratios increased up to day 7 and decreased until cycle 2, suggesting that MIC-1 changes were dependent on AMG 232 exposure.

### Efficacy

**Fig. 3:**
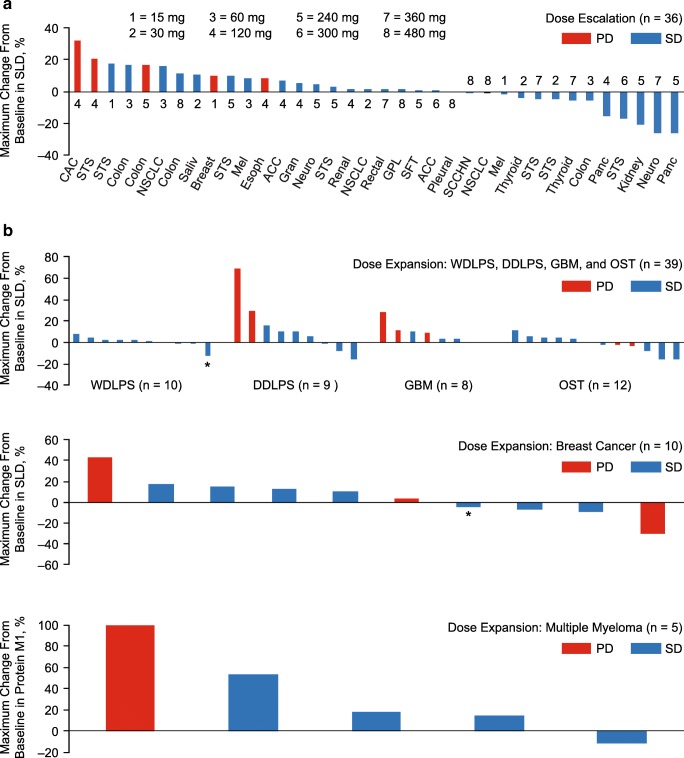
Best change from baseline in the sum of longest diameters of target lesions in the dose escalation (**a**) and in the dose expansion (**b**). ACC, adenoid cystic carcinoma; CAC, cholangiocarcinoma; Chondro, chondrosarcoma; DDLPS, dedifferentiated liposarcoma; Esoph, esophageal; GBM, glioblastoma multiforme; GPL, glandular parotis left; Gran, granular cell tumor; Leio, leiomyosarcoma; Mel, melanoma; Neuro, neuroendocrine; NSCLC, non–small-cell lung cancer; OST, other solid tumor; Panc, pancreatic; Pleural, pleural mesothelioma; Saliv, salivary gland; SCCHN, squamous cell carcinoma of the head and neck; SFT, solitary fibrous tumor; STS, soft tissue sarcoma; WDLPS, well differentiated liposarcoma. *Patient had partial response per central review

Imaging for the local evaluation of tumor response was available for 38 of 39 patients in the dose escalation and 60 of 68 patients in the dose expansion. One patient in the dose escalation had no postbaseline imaging due to an AE. Eight patients in the dose expansion had no postbaseline imaging due to clinical disease progression (*n* = 3), AEs (n = 3), and patient request (*n* = 2). By local evaluation, no objective responses were observed. Per central evaluation, three patients (4%) with WDLPS, squamous cell carcinoma, and breast cancer had unconfirmed partial responses with durations of 2.4, 0.1, and 2.0 months, respectively. In the dose escalation, 31 (80%) patients had stable disease and five (13%) had progressive disease (Fig. 3a). Based on evaluation of non-target lesions, 2 (5%) patients in the dose escalation had non − complete response/non − progressive disease. Overall, 45 (66%) patients in the dose expansion had stable disease and 15 (22%) had progressive disease (Fig. 3b). Stable disease as a best result in the dose expansion was observed among 10 of 10 patients with WDLPS, 7 of 10 with DDLPS, six of 10 with GBM, 10 of 16 with other solid tumors, 10 of 12 with breast cancer, and 5 of 10 with multiple myeloma.

**Fig. 4:**
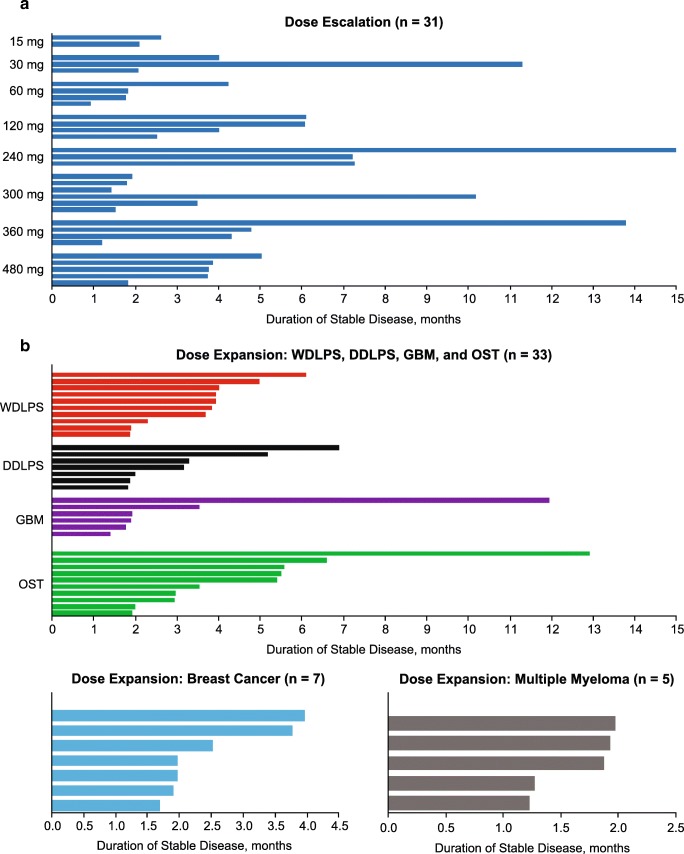
Duration of stable disease in the dose escalation (**a**) and in the dose expansion (**b**). DDLPS, dedifferentiated liposarcoma; GBM, glioblastoma multiforme; OST, other solid tumor; WDLPS, well differentiated liposarcoma

Duration of stable disease in the dose escalation and across histologies in the dose expansion is summarized in Fig. 4. The median duration of stable disease in the dose escalation was 3.1 months (range, 0.7–22.4 mo). In the dose expansion, the overall median duration of stable disease was 2.0 months (range, 0.5–12.9) overall, 3.9 months (range 1.9–6.1) among patients with WDLPS, 2.0 months (0.9–6.9) among patients with DDLPS, 1.8 months (0.5–11.9) among patients with GBM, 3.3 months (0.9–12.9) among patients with other solid tumors, 2.0 months (0.9–4.0) among patients with ER + PR+ breast cancer, and 1.4 months (0.9–3.8) among those with ER + PR– breast cancer.

## Discussion

In this first-in-human study, AMG 232 was generally well tolerated up to 240 mg. AMG 232 doses up to 480 mg QD for seven days in a 21-day cycle were assessed; however, the MTD was not reached. Three patients had DLTs, two with thrombocytopenia and one with neutropenia. Based on these DLTs and other cytopenias outside of the DLT evaluation window, AMG 232 240 mg was selected as the highest safe and tolerable dose for evaluation in the dose expansion. In both phases of the study, AMG 232–related AEs were generally mild to moderate, with the most frequently occurring AEs being diarrhea, nausea, vomiting, fatigue, decreased appetite, and anemia. Among the AEs resulting in treatment discontinuation, thrombocytopenia, neutropenia, and gastrointestinal toxicity were the most frequent. These results are consistent with other clinical studies of MDM2 inhibitors, in which myelosuppression and gastrointestinal toxicity have been reported [18, 19, 28–33]. In this study, five patients died due to disease progression while on the study.

Following QD treatment for seven days in a 21-day cycle, AMG 232 plasma concentrations increased generally dose proportionally, and across all patients, the mean AUC accumulation ratio between days 1 and 7 was less than 2-fold. Overall, AMG 232 exhibited an acceptable pharmacokinetic profile in this population. Furthermore, dose-dependent increases in serum MIC-1 levels from baseline to day 15 of treatment indicated p53 pathway activation, consistent with previous studies of MDM2 inhibitors [19–21].

One of the objectives of the dose expansion was to assess the antitumor activity of AMG 232 among patients not only with P53WT tumors but also among those with *MDM2* amplification and *MDM2* overexpression, which is common in liposarcomas, GBM, breast cancer, and multiple myeloma [5–11] Per local evaluation, no objective response were observed. Per central evaluation, 3 patients had partial responses. Overall, stable disease was observed in 45 of 68 (66%) patients in the dose expansion, including all 10 patients with WDLPS, 7 of 10 with DDLPS, 6 of 10 with GBM, 10 of 16 with other solid tumors, 7 of 12 with breast cancer, and 5 of 10 with multiple myeloma. Stable disease was observed among patients with WDLPS (median, 3.9 months; range, 1.9–6.1), which is naturally an indolent disease, followed by other solid tumors (median, 3.3 months; range, 1.9–12.9), DDLPS (median, 2.0 months; range, 1.8–.6.9), GBM (median, 1.8 months; range, 1.4–11.9), breast cancer (median, 2.0 months; range, 1.7–4.0), and multiple myeloma (median, 1.0 months; range, 1.2–2.0). Our results are consistent with those of previous reports demonstrating limited clinical activity with HDM2/MDM2 inhibitors [18, 20, 31, 34–36]. Because the development of *TP53* mutations may contribute to the development of resistance to treatment with HDM2/MDM2 inhibitors, investigation of HDM2/MDM2 inhibitors in combination with other agents may be appropriate [34].

In conclusion, AMG 232 showed acceptable safety, dose-proportional pharmacokinetics, and on-target activity as monotherapy administered at oral doses up to 240 mg QD on days 1–7 per 3-week cycle in patients with P53WT solid tumors. No responses were observed per local evaluation but 3 patients had unconfirmed partial responses per central evaluation. Stable disease was observed among patients regardless of *MDM2* amplification or overexpression. Future evaluation of AMG 232 in hematologic malignancies and solid tumors should be considered.
